# Remarks upon the Subject of Infanticide

**Published:** 1833-01

**Authors:** Alfred S. Taylor

**Affiliations:** Lecturer on Medical Jurisprudence, &c. in Guy's Hospital.


THE LONDON

### Medical and Physical Journal.

407, VOL. LXIX.] JANUARY 1833. [79, *New Series*.

"For many fortunate discoveries in medicine, and for the detection of numerous errors, the world is indebted to the rapid circulation of Monthly Journals; and there never existed any work to which the Faculty in Europe and America, were under deeper obligations than to the 'Medical and Physical Journal of London,' now forming a long but an invaluable series."—RUSH.

### ORIGINAL PAPERS.

### INFANTICIDE.

*Remarks upon the Subject of Infanticide.* By ALFRED S. TAYLOR, Esq., Lecturer on Medical Jurisprudence, &c. in Guy's Hospital.

(Continued from page 367 of the last volume.)

IT has been hitherto my object to determine the extent to which the objection on the ground of putrefaction in the lungs, may influence the results obtained by the employment of the *Docimasia pulmonaris*, or hydrostatic test, in cases of suspected Infanticide. How far I may have succeeded in this, it will be for the reader to determine; but I cannot dismiss this "objection" without endeavouring to expose still further its utter untenableness. For this purpose I will take two supposed cases, upon which the medical jurist may be consulted. The principal facts regarding the generation of air in the lungs during the process of putrefaction having been already stated, it will be unnecessary to revert to them.

In the first of these cases, I will suppose that putrefaction has taken place to a very great extent; that not only the lungs, but every other organ in the body, will float, when immersed in water; that the canalis arteriosus is pervious throughout; and that the foramen ovale is open.* Here, then, the medical witness is called upon to determine whether that child has respired or not. Upon examining the lungs, the air may be observed, as described by Dr. Hunter, dispersed in large vesicles along the fissures and between the component lobuli; yet still, when these organs are submitted to violent compression, it may be found impossible to deprive

* The reasons for observing the state of these two parts will be more fully explained in the sequel; but I may now observe, that, soon after the establishment of respiration, the diameter of the one becomes diminished, and the other contracted.

them of their buoyancy: for vesicles of air may also exist in the internal parenchymatous structure of the organs, and effectually resist all attempts at expulsion. What decision, then, can he come to upon this case? If he refer to the majority of writers on the subject, he will find it stated, that air, generated by putrefaction, is formed only on the surface of the organs; and that, when thus formed, it may be readily forced out by compression. But it will be necessary for him to inquire, previously to founding an opinion upon these statements, how far they correspond with ascertained facts. That air is commonly found upon the surface of the lungs, where putrefaction has taken place, is undoubted; but that it always is found in this situation under these circumstances, or that its distribution throughout the structure of these organs is to be taken as a certain criterion of its origin, is more than doubtful. I presume that the same inference will be allowed to hold with the liver as with the fœtal lungs, where decomposition has taken place to any extent; and I have repeatedly had occasion to remark, in such cases, that central portions of this organ would float as readily as those which were taken from the margin.* Hence the idea of resting our decision upon the particular situation of the air must be abandoned; at least in such an extreme case as *that* which I am now supposing.

The question whether air generated in the lungs by putrefaction can be always forced out by compression, has been already in part considered. On this point Orfila, who is, generally speaking, a most accurate observer, says, "On exprimera les poumons entre les doigts, et l'on verra en les plaçant sur l'eau qu'ils se précipitent dans les cas de putrefaction; tandis qu'ils continuent a surnager s'il y a eu respiration; en effet les gaz, développés pendant la fermentation putride, sont logés dans le tissu lamineux, qui separe les cellules bronchiques et le plus souvent entre la plêure et les poumons: *ou la plus légère pression suffit pour les degager;* tandis que l'air atmospherique, qui distend les poumons, pendant la respiration, occupe les cellules bronchiques et ne peut en être expulsé qu'avec *la plus grande difficulté.*"† Other

* Air is certainly necessary to the process of putrefactive fermentation; but this, as is well known, is not the only condition. The gaseous elements of which our organs are composed may react upon each other, and produce other gaseous compounds in the central part of an organ, as well as in the exterior. In the latter case, they will distend the investing membranes, and form conspicuous vesicles on the surface; while, in the former, the pressure of surrounding parts may for a time suspend their development, although it cannot prevent their formation.

† Orfila, Médécine Legale, tome i. p. 358.

authors have expressed themselves in the same manner; but the cases from which these conclusions are derived have been generally those in which putrefaction had not very far advanced. No individual has hitherto asserted it to be impossible that putrefied air should ever assume a disposition in the lungs similar to that in which respired air is commonly met with; indeed, it must be admitted that there is nothing in the nature of things to forbid us believing that such may occasionally be the case. If so, what becomes of this pretended criterion to be derived from the expulsion of the air by compression? It must, in so far as *these cases* are concerned, fall to the ground; and, probably, the dogmatical assertions to the contrary have tended in no slight degree to increase that scepticism in the profession relative to the utility of the "test," the foundation of which had already been laid by Dr. Hunter. The impossibility of expelling the air from the lungs, as I stated in my former paper, does not prove that it had really been introduced by respiration, where putrefaction has proceeded to its most extreme degree; for a sufficient number of cases has not yet been collected to warrant any such presumption. Those medical jurists, therefore, who dwell upon the certainty of forming a decision by the simple compression of the pulmonary organs, must be understood as referring to cases in which putrefaction has only commenced.

Here then, having reduced the question to its most simple state, we will now see upon what basis the medical jurist is to rest his judgment. The history of the case may be unknown or purposely concealed, and some female may be awaiting the decision of a coroner's court, as the suspected murderer. The surgeon is called upon to decide whether the child had been born alive; which, as I have stated, in the *legal* sense, signifies to have respired;* and he, taking into

* It is remarkable that our law is so frequently contradictory to itself on many points relating to physiological science. Thus, in cases of infanticide, a child is not considered to have lived, unless it can be proved that it has performed the act of respiration; while, in cases relative to the succession, or to the inheritance of property, the most trifling indication of the presence of the vital principle, such as a motion of the lips or a quivering of a muscle, is considered sufficient to prove that the child was born living. In disputed cases of *tenancy by the courtesy*, these nice determinations are very frequently met with. A curious case of this kind was decided in the Court of Exchequer, at Westminster, in the year 1806, in which a Mr. Fishe was plaintiff: it was here determined by the court, that, although the child was stillborn, a tremulous motion of its lips, witnessed by the nurse immediately after its birth, was sufficient to prove that it had lived, and therefore, according to law, the estate reverted to the plaintiff. Now, if a question of infanticide had arisen respecting such a child, the decision of our legal authorities would have been, that it was born dead, and therefore that it could not have been murdered!

consideration all the circumstances above described, cannot but answer, "that no certain opinion could be given; for the putrefactive process had completely destroyed those appearances which might have otherwise afforded a satisfactory solution." Even supposing that his prudence were to forsake him, and he were positively to affirm that the child had lived and breathed after birth, because he had not been able to force out the air from the lungs, what would be the result? His affirmation would only go to warrant the presumption that the child had *not been born dead;* but this alone would not establish the fact of murder against the mother; for she is not called upon to prove that she did not murder the child. Now, supposing that prejudice has operated on the mind of the practitioner, as it but too often does on the minds of the vulgar, he may have been hastily led to depose to the probability of this child having been murdered; but it will be difficult to understand whence he is to draw his proofs, when the female is placed on her trial; for, in order to establish the commission of the offence, he must first shew that it was impossible the child could have died from any other cause than from violence or neglect on the part of the suspected individual.

It is doubtless from a want of proper attention to this point that many of the acquittals, which take place on trials for infanticide, are to be attributed. The mere proof that the child has lived, is considered to be sufficient to establish the fact of its having been murdered; and a coroner, in nine cases out of ten, will, upon such evidence, commit a female for trial; although it is manifestly as absurd to draw such a conclusion, without strong corroborating circumstances with regard to the body of a child, as with regard to the body of an adult that may be found dead on the highway. It is virtually denying that a child may perish from natural causes, which, as every obstetric practitioner knows, are sufficiently numerous, and are continually operating to destroy a great proportion of those children against whose mothers not a suspicion is ever breathed.

At the Old Bailey sessions, in July last, a woman was indicted for the murder of her child, which was found dead in a watercloset, to which it was proved she was in the habit of retiring. Before the coroner, it had been stated by the medical practitioner who conducted the investigation, that the child had lived to respire. According to the statement of the female on the trial, she was suddenly and unexpectedly delivered on the spot, when the child fell and was suffocated. There was no evidence to impeach her integrity, nor was there any reasonable ground for suspecting that she had committed the offence laid to her charge. The medical witness, upon being questioned by the court respecting the possibility of delivery taking place in such a situation, and rendering the mother incapable of affording the requisite assistance to the child, was, of course, compelled to answer in the affirmative; and the prisoner was instantly acquitted.

If this case had occurred in a distant part of the country, the female might have suffered eight months' imprisonment upon a groundless charge; and, as it was, she was placed like a felon on her trial, to the great injury of her reputation. It is not for me to decide whether, in this case, the fault attached more to the coroner than to the medical witness; but it is evident, if the coroner had done his duty in putting certain questions to the latter, which a proper knowledge of the subject would have suggested, the female would not have been unnecessarily brought to disgrace and ignominy.*

To return from this slight digression. The reader will perceive that two ways are open to the practitioner, to decide upon so doubtful a case as that which I have imagined. If he assert that medical science is incapable of affording a solution of the question at issue, as he undoubtedly would if he had bestowed a proper attention on the subject, he is not exposing the female to any risk of a prosecution on the charge of murder; nor is he throwing discredit upon the employment of the hydrostatic test. Indeed, to maintain the latter would be a downright solecism; for it would be equivalent to maintaining that, because we cannot *always* make use of certain means of investigation, we are never to trust to them; a proposition to which few rational practitioners would be disposed to accede. When we speak of the value of employing certain tests for mineral poisons, we do not call to mind the number of cases in which they have failed to give satisfactory results, because we know that there are numerous conditions which, in spite of all human ingenuity, will cause them to fail: but we dwell upon the cases in which they have been known to succeed, and where it is possible, endeavour to avoid those circumstances to which their failure is to be attributed. This is precisely the course to be pursued in in-

* It has been recently decided in the House of Commons, that a knowledge of medical jurisprudence is altogether *unnecessary* to the coroner, on the ground that the study of the science would occupy too much of his time, and distract him from his immediate duties. It would be a curious question to determine, whether the time lost by the coroner in this study would not be so much time saved from unnecessary imprisonment to those who have to suffer from his ignorance of the subject?

vestigations relative to infanticide; and to contend that, because, in either case, these investigations do not invariably lead to *certain* conclusions, we ought to abandon those means which rational experience points out, would be the height of absurdity in reasoning. We should, indeed, be acting the part of the man described by the celebrated Locke, "who would not use his legs to walk, because he had not wings to fly with."

But I have presumed, on the other hand, that a practitioner may be found who will swear that the child has lived to respire, and inform the court that he has come to this conclusion from the employment of the hydrostatic test. The test is at once denounced, and the conclusion derived from it rejected; but it is not for a moment supposed that any error may have arisen from the want of judgment or from incapability on the part of the individual who has employed it. On the contrary, the hostility of all is immediately turned against the means which have been used; and the practitioner finds himself unable to encounter the cross-examination of counsel. The result is, that the charge is dismissed; but is it possible that such a result can be attributed to the failure of the hydrostatic test? Is it not proper that we should first satisfy ourselves whether unjustifiable inferences have not been drawn from its employment, before we proceed to condemn it as useless? Although the answers to these questions are obvious and satisfactory, there are very few instances in which they have been asked, and, therefore, the answers have escaped observation. Now such cases form a very large majority of those which come before our courts of justice, and hence the repeated failure of what has been regarded as infallible evidence has led practitioners to fly into the opposite extreme; a circumstance which has brought the Docimasia Pulmonaris into greater disrepute than all the arguments of its most violent opponents.

From what has been stated of the case which I have supposed the practitioner to meet with, it will be at once seen that he is justified, neither by experiment nor by observation, in drawing the above inference. If, therefore, in such a case, he assert that the hydrostatic test proves that respiration has taken place after birth, he is asserting more than he is warranted in doing, and, of course, must take the whole responsibility of the result upon himself. But in spite of this obvious conclusion, the principle upon which we have been acting for many years is precisely the reverse. We have universally condemned the means by which the conclusion has been arrived at, and we have not fairly examined the grounds upon which it was founded. If such a course had been pursued with regard to the uncertainty of the tests employed in the analysis of poisons, the science of toxicology would now be, what it was but a few years since, a mere shadow: and as, in every subject connected with medicine, old and unfounded prejudices are rapidly sinking before the light of experience, we may hope that this branch of medical evidence will not long remain in its present unsatisfactory condition.

We have here, then, taken the most extreme view of the "objection" on the ground of putrefaction; but we will now consider that the process has not advanced so far as has been represented. In cases of this description it will commonly be found, where putrefaction has extended to the lungs, that air is collected beneath the pleura in large vesicles, as also that the central portions of the organ are perfectly destitute of air, and therefore sink: although, those taken from near the surface may have sufficient buoyancy to enable them to float when immersed in water.* It is also found that, on compression, these last-named portions may be deprived of their air, and be made to sink. The *canalis arteriosus* must be found pervious, and of equal diameter throughout; the *foramen ovale* open; and the skin of the umbilical cord perfectly continuous with that of the abdomen, presenting around the margin of its insertion no appearance of inflammation or ulceration. What is the conclusion which the medical jurist is justified in drawing from these facts? I presume that not even the most sceptical would hesitate to admit that they clearly prove that the child in question had not performed the act of respiration, and, therefore, according to law, that it had not lived. It might be said the child may have respired, and yet the quantity of air introduced have been so small† as to become readily forced out by compression. This objection, however, could only create any difficulty in the most advanced cases of putrefaction, where the air, generated by this process, is retained within the central structure of these organs; for, in the case of which I am now speaking, the air is supposed to be distributed, as in incipient putrefaction, in large vesicles over the surface of the pulmonary structure, and not in the central portions. Now, if it had been introduced by respiration, it will be found, previous to submitting the divided portions of the lung to compression,

* I here presume that the lungs are healthy; for it is undoubtedly true that some diseases may harden their structure, after the establishment of respiration, to such an extent as to deprive them of their usual buoyancy.

† Vide page 366.

that the air is not diffused in large vesicles over the surface, but contained in the minute cells of the internal portions; and, moreover, that the condition of buoyancy is precisely reversed; for those taken from the surface will sink, while the central portions will float. Therefore, it will be perceived, even allowing it to be possible that respired air can be forced out by compression, which is of itself doubtful, we have still in our hands means of determining the question.

Those who have argued against the hydrostatic test, as being liable to lead to the condemnation of the mother, when innocent, in bringing forward the above objection have proved too much: for it will be immediately apparent that, in cases of feeble respiration, if we can expel the air, as in cases of incipient putrefaction, without for the present dwelling upon its precise situation, we are led to draw the inference that a child, which may have merely made a single gasp on its entrance into the world, was born dead; in which case it is clear that the mother, so far from incurring a risk of her life, will be acquitted.

It is absolutely necessary, in order to unravel the intricacies involved in this subject, to pursue it through all its ramifications, and to combat the objections as they arise. It is my object, however I may act up to it, to shew not only the advantages, but the disadvantages, which are likely to result from employing this test as a means of investigation; and I am as little anxious to exaggerate its merits, as I am to conceal its defects. Nevertheless, it is impossible to allow idle or unmeaning objections to obscure those points which are really useful, or to give strength to those which are *apparently* defective, when the whole may be proved to be groundless, by adopting a proper method of examination.

Sufficient, perhaps, has been said to develop the nature of the obstacles which the process of putrefaction is likely to offer to the employment of the Docimasia Pulmonaris, as well as to point out the means by which those obstacles may be removed. But, as there is still a collateral proof, which lies open to the medical witness, it will be necessary to determine how far, in the absence of all other evidence, he is justified in relying on *this*. I allude to the test of Ploucquet.

In order to understand the nature of this test, we must bear in mind the changes which take place in the lungs by the establishment of the process of respiration. Previous to birth, the pulmonary vessels circulate but a very small quantity of blood through the respiratory organs, the greater portion of the contents of the right ventricle passing directly through the *canalis arteriosus* into the *aorta.** Now, in consequence of the great quantity of air introduced into the air cells of the lungs by the act of respiration, and in consequence of the greater buoyancy which these organs thereby acquire, we are apt to form an erroneous judgment respecting the change which really takes place; we are apt to conceive that they become of less specific gravity than they were originally. So far from this being the case, however, their weight becomes nearly doubled; and this increase of weight they derive from the circumstance of a much larger quantity of blood becoming circulated through them. The increased buoyancy, therefore, is owing to a simple mechanical cause, to the retention of a vast quantity of air in their retiform structure; and so well distributed is this air, that it is enabled to counteract the increase of weight which is brought about by the alteration in the course of the circulation.

The test recommended by Ploucquet is founded on these simple principles. We are to determine the weight of the body of the child, then to remove the lungs from the chest, and, having weighed them, to ascertain what proportion the weight of the lungs bears to the weight of the body. Whatever that proportion may be, it is clear, from what has been above stated, that it will be nearly twice as great in fœtuses which have respired as it is in those which are still-born. Of course, it was necessary that some standard should be fixed by which the result, in either case, might be compared; and accordingly Ploucquet performed some experiments on the subject, and from these he deduced the following proportions: 1st. That where respiration had not taken place, the weight of the lungs was, to the weight of the body, in the ratio of 1 to 70. 2d, That where the act of respiration had been completely performed, the weight of the lungs was to the weight of the body in the ratio of 1 to 35.†

It has been objected to these inferences of Ploucquet's, that his experiments were not sufficiently numerous, and that the average was fixed as much too high in the one case as it was

* Professor Foderé and others have looked upon the presence of blood in the pulmonary vessels as affording most certain evidence that respiration has taken place, and its absence they have regarded as tending to prove the contrary. Of four stillborn subjects, which I have successively examined, I have found semi-coagulated blood in the pulmonary vessels; and in several, in which respiration had taken place, none was found, owing to accidental causes. This will suffice to show how little reliance is to be placed upon such an appearance.

† The weight of the air introduced into the lungs by the process of respiration cannot be considered as adding to the absolute weight of these organs, since they are weighed in air.

too low in the other. It has certainly been proved by the observations of several distinguished physiologists, since his time, that his data were not well founded. Professors SCHMITT, of Vienna, and CHAUSSIER, of Paris, in order to investigate the truth of his positions, undertook a series of experiments on the subject, and the results at which they arrived are, that the body of a fœtus which has respired has rarely so low a ratio as 50 to 1, taken with the lungs; while the body of a fœtus which has not respired generally bears a lower ratio to the weight of the lungs than as 35 to 1.*

During the last summer, I employed this test in four cases on stillborn subjects, and the proportions which I obtained will be seen below.

| Weight of the Lungs. | Weight of the Body. | Proportion. |
|---|---|---|
| Grs 640 | Grs. 38,400 | 1 to 60 |
| 687 | 33,847 | 1 to 53 |
| 300 | 14,400 | 1 to 48 |
| 1950 | 41,760 | 1 to 21. |

It will be observed, that the three first of these cases correspond very nearly with that proportion which previous calculations had given; but the last is to be regarded as a most extraordinary exception to the rule. The weight of the body of this fœtus was about the common average; and, therefore, the extreme preponderance is to be set down to the lungs; for it will be seen by the table that they were three times the weight of those organs in the two first cases, and six times the weight of those in No. 3. In fact, their enormous size did not fail to strike every observer, and, from their appearance, it was at once concluded that they would bear a considerable proportion to the weight of the body. Such instances, however, of so great a departure from the standard, are to be looked upon as extremely rare, and as altogether insufficient to invalidate the general application of the test.

It cannot be denied that the body of the fœtus is liable to vary in weight, according to the greater or less quantity of adipose substance about it, as also according to its length and its sex: but these variations are confined within a small limit, and the knowledge of the causes from which they may proceed will be sufficient to remove the errors that might otherwise possibly creep into the calculation. It is also worthy of note, that the ratio is exceedingly constant with regard to the fœtus in utero up to the full period of gestation, which shews that there is certainly a strict relation between

* For the tables which these physiologists have drawn up relative to this subject, vide Orfila, Traité de Médecine Légale, tome i. p. 348.

the development of parts; and if it be not always discovered after the establishment of the respiratory process, we must endeavour to make allowances for its absence, and frame our decisions accordingly.

Now, by applying the test of Ploucquet to the cases which we have been considering, we shall, in a great majority of instances, be able to corroborate the presumption which we may have drawn from other facts. Supposing, in either of the cases in which putrefaction has taken place, that we find the weight of the lungs to be, to the weight of the body, in the ratio of about 1 to 50, we are certainly confirmed in our suspicion that respiration had *not* been performed; for the air generated by putrefaction cannot give to these organs any additional weight. This, as I have already stated, must be derived solely from the altered course of the circulation. But, it may be argued, the ratio may turn out to be higher than 1 to 50; it may be, for example, from 1 to 30 or 40, and thus closely approximate to the results obtained by the experiments on fœtuses which *have respired.* This, it is true, is converting the exception into the rule; but, nevertheless, it shall be admitted, for the sake of argument, that a higher ratio is obtained than that which I have above given. What, therefore, is to be the course which the medical jurist ought to pursue in this dilemma? He has, I conceive, simply to consider on which side the error may probably lie, or whether both results may not be reconciled without any great violation of fact or reasoning. In the cases which I have supposed, the latter plan may be safely followed, since a high ratio, obtained by the test of Ploucquet, may by possibility occur in a fœtus which has not respired, as well as in one which has respired; and, although a low ratio might be strongly corroborative of our original presumptions, a high ratio would not militate against it.

Therefore, if, in the examination of a child suspected to have been born alive, we find that the lungs will float when immersed in water, but that they may be deprived of their buoyancy by moderate compression; as also that the air to which that buoyancy is owing is confined to the surface in the form of large vesicles, and not diffused uniformly throughout the cellular parenchyma of the organs: it will matter but little whether their weight be, to that of the body, in the ratio of 1 to 50 or of 1 to 35. In either case, the practitioner will be justified in affirming that the suspicion of the child having respired is unfounded. If he obtain the lower ratio, it will be so far confirmatory of the correctness of his decision, as it corresponds with the general rule; if the higher, it cannot undermine his decision, since it may readily fall within the class of exceptions.

With this, then, I think the objection raised by Dr. Hunter to the employment of the hydrostatic test, on the ground that the process of putrefaction might generate air in the lungs, and might give results precisely similar, may be altogether dismissed. If any doubt should remain in the mind of the reader, let him deliberately examine the disputed question in all its bearings: let him examine the evidence which has been given on trials for infanticide, where this question has arisen: and I do not doubt he will come to the conclusion that the "test" has been most improperly condemned, either from its misapplication by the witness or from the mistaken views which may have been entertained respecting the results to be obtained from its employment.

Let us now turn our attention to another "objection" which has been brought into notice by Dr. Hunter's "Tract." In this it is supposed that a newly-born child is found under suspicious circumstances, without any appearances of putrefaction on its body. The practitioner examines the body; he finds that the organs are generally healthy, that the lungs are distended with air, and that, when immersed in water, they immediately rise to the surface, where they remain. Is he justified in pronouncing, under these circumstances, that this child has respired? or are there any artificial means by which the appearances above described may be produced? It will be seen by the following extract from Dr. Hunter's Treatise* that these appearances may be artificially stimulated. Thus, he says, "If the air in the lungs be found to be contained in the natural air vesicles, and to have the appearance of air received into them by breathing, let us next find out if that air was not perhaps blown into the lungs after the death of the infant. It is so generally known that a child, born apparently dead, may be brought to life by inflating its lungs, that the mother herself or some other person might have tried the experiment.† It might even have been done with a most

* On the Uncertainty of the Signs of Murder, &c., p. 29.

† The experiments of M. Leroy d'Etiolles, the results of which were communicated to the Institute in the year 1826, have thrown a new light upon this subject. His experiments were performed upon young rabbits, puppies, and lambs; and he found that, where the process of inflating the lungs artificially was not conducted with the greatest caution, death almost instantaneously ensued. This result could scarcely be attributed to a rupture of the bronchial cells, for this was but seldom observed to take place. We may hence presume that the very means which the vulgar consider necessary to restore animation may be sufficient to prevent it, unless used with the greatest care.

diabolical intention of bringing about the condemnation of the mother."

In making this statement, the Doctor does not appear to have appreciated the difficulty of performing this experiment, and, from the manner in which he has stated it, it seems doubtful whether he ever made the trial. It was, however, well calculated for the purpose intended, namely, to throw uncertainty upon the means employed in the investigation; and, at the time it was first promulgated to the world, it was deemed an unanswerable objection. We will, however, see how the case stands now.

Where the lungs are removed from the body, and a pipe is introduced into the trachea, there will be no difficulty whatever in giving to the lungs the colour, appearance, and physical properties which they are supposed to derive exclusively from the act of respiration. But the conditions are far otherwise in the case of the newly-born child; and those who try the experiment will find that there is generally considerable difficulty in causing even a moderate distention of these organs by the application of the mouth to the mouth of the child, even after long-continued efforts. This must certainly be presumed to be the more common mode of its introduction, since it cannot be admitted that the mothers of children, likely to fall under the class of cases imagined by Dr. Hunter, should always be provided with tubes for the express purpose of inflating the lungs. But the question now offers itself, how many of such females are there who can even be supposed to know any thing about an experiment of this nature, much less to be capable of undertaking it at a time when the greatest exhaustion and weakness must necessarily fall upon them? It is, moreover, an experiment which must not be delayed, or otherwise it cannot be performed with any rational hope of success. I presume, of course, that the female is alone and unassisted, and that it is her first pregnancy; for these are the cases which chiefly come before our courts of law. Indeed, to admit that any individuals should be present or assisting her would, in the majority of cases, do away with the substance of the objection; for, under these circumstances, a charge of infanticide is rarely, if ever, brought forward, much less substantiated. Dr. Hunter, it is true, says, "some other person might have tried the experiment:" but let the history of infanticides be read, and it will be found that the presumption is wholly unwarrantable. After very diligent search, I have not been able to meet with a single case which would afford the least ground for making such an assertion. Is it ever put forward in her defence by the female, that the buoyancy of the lungs of the child was owing to her having inflated them artificially? Is there a single instance in which this artificial inflation has ever exposed the life of the mother, or of the supposed accessary, to jeopardy? and, if not, are we compelled to invent for her a defence? Are we compelled to allow that she can execute that which it requires caution, presence of mind, and strength to execute, when we know that such admissions are contrary to all reasonable probability? But although, in one part of his work, the Doctor allows that a female may have sufficient presence of mind and strength to perform this act, in another,* where his inference is drawn for a very different purpose, he supposes that a woman, at the time of her delivery, cannot be under the direction of a "calm and deliberate mind;" but that, on the contrary, any irrational conduct on her part must be justified by the "violently agitated state of her mind," and by the "conflict of passions and terror" under which she must be labouring.

It will doubtless be evident to every reader, that the author of the "Uncertainty of the Signs of Murder" would have better served the purpose for which he wrote, had he first ascertained whether this pretended "objection" had done the mischief which his manner of stating it would lead the world to suppose. As it has stood since his time, it may have served the purpose of enabling a barrister to make a skilful defence for his client, as well as of spreading through both the medical and legal professions a feeling of prejudice against the ordinary means of research; and, until its fallacies be thoroughly exposed, it may still continue to obscure the truth, and to obstruct the stream of justice. The slightest reflection upon the nature of this objection will inform him who has attended to probable or suspected cases of infanticide, that, if it were admitted to have its full value in our courts of justice, it would be most admirably fitted to bring about *the acquittal of the guilty:* but I hope to be able to shew that, even allowing of the existence of this, to my mind, greatest of improbabilities, we are yet in possession of means by which the artful ingenuity of those who have offended against the laws of God and man may be rendered capable of detection and exposure. The assertion that the experiment may have been performed by another party, with the intention of bringing about the condemnation of the mother, is wholly unsupported by fact or reasoning: for it is hardly necessary again to state that the mere circumstance of the

* Page 21.

floating of the lungs of a child in water does not prove that it has been murdered by its mother: it merely demonstrates that the lungs are distended with air; but we must determine how that air entered into the organs, before we can even affirm that the child has *respired;* and we must render it certain that the child has not died from *natural causes,* before we can affirm that it has been *murdered!*

(To be continued.)

35, *Great Marlborough street; November* 1832.

---

